# Supplementary material for: Return of Participants’ Incidental Genetic Research Findings: Experience from a Case-Control Study of Asthma in an American Indian Community
Source: J Pers Med. 2023 Sep 20;13(9):1407. doi: 10.3390/jpm13091407 (PMC10532458; doi:10.3390/jpm13091407)
Supplement: Supplementary file 1 [file jpm-13-01407-s001.zip › jpm-2558581-supplementary.pdf]

5/25/21

Dear XXX,

I'm very glad I had a chance to visit with you again last Tuesday, and happy that you were able to get those tests done with good results. We were able to get copies of your records from Dr. XXX's office (thanks to the release form that you signed), and I reviewed them in some detail. In Dr. XXX's notes it mentioned that he did not get a copy of the Mayo Clinic results, so I sent him a copy and gave him a little more detail on what this genetic change means in some people. I also made sure he was aware of the problems you had when …to the hospital… and you had to meet the ambulance part way there. I wasn't sure if he had any of those records or was aware of this episode, but maybe you told him during your visit.

The thickness of your heart muscle was normal and that is one of the main things that begins to change when people with this genetic difference have trouble. So this is very good news, although these changes don't often happen at your age, so I think you will want to have one of these echocardiograms done every once in a while (maybe every 5 or 10 years), but your doctors should be able to tell you how often. I did see mention of the tiny hole between the upper two chambers of your heart. This hole is normally present when you are born, but is supposed to close completely within a few days. The size of this in your case seems to be small enough that it shouldn't bother you. Your heart rhythm seems to be good also, although just a tiny bit faster at rest than usual. The medication (metoprolol) that he gave you should help with that.

Your blood pressure was very good during your visit, so that is another great sign; but please make sure you have it checked from time to time, probably at least once a year. Of course it will help your heart a lot to do the usual smart things, like not smoking, keeping your weight normal, eating a healthy diet and getting regular exercise.

It is important for you to understand that this genetic change that you inherited doesn't mean 100% that you will develop heart trouble. In the other people with this exact same change, about half end up with heart wall thickening and other problems, but the other half seem to remain normal. I know you have discussed these results with your …sibling… and XXX doesn't want to go through the same testing; but I hope XXX comes to understand that even though you are siblings and we know XXX has the same genetic change that you do, how XXX body reacts to this might be different. Please let XXX know that if XXX changes XXX mind, we are still willing to help XXX get the testing that would be useful. At the very least, encourage XXX to have XXX blood pressure checked regularly, and do those other smart, heart-healthy things.

As we mentioned, we could arrange testing for your …parents… as well; but since that time, we were able to check on some of the same tests that were done for your one of your parents (through the Strong Heart Study) and we think it is extremely unlikely that XXX could be the one that gave you and your sibling this genetic change. Therefore, it likely came from your other parent, and we are also willing to get testing for

XXX. I also know that you have pretty limited contact with your …other parent… and it is entirely up to you as to what you wish to share with XXX.

So please keep my contact information and don't be shy about letting me know if you have any further questions that I can help with. Wishing you the best.

Sincerely,

Lyle Best, MD

1935 118th Ave NW

Watford City, ND 58854

701-842-6770 or 701-871-7345

lbest@restel.com
